# Suggested Role for G4 DNA in Recombinational Switching at the Antigenic Variation Locus of the Lyme Disease Spirochete

**DOI:** 10.1371/journal.pone.0057792

**Published:** 2013-02-28

**Authors:** Rupali Walia, George Chaconas

**Affiliations:** 1 Department of Biochemistry & Molecular Biology, Snyder Institute for Chronic Diseases, University of Calgary, Calgary, Alberta, Canada; 2 Department of Microbiology & Infectious Diseases, Snyder Institute for Chronic Diseases, University of Calgary, Calgary, Alberta, Canada; University of Kentucky College of Medicine, United States of America

## Abstract

Antigenic variation through targeted DNA rearrangements provides a powerful diversity generating mechanism that allows a variety of pathogens to stay one step ahead of acquired immunity in their hosts. The Lyme disease spirochete encodes such a system that is required for persistent infection. The *vls* locus, carried on a 29 kb linear plasmid (lp28-1) in the type strain B31, carries 15 silent cassettes from which information is unidirectionally transferred into the expression locus, *vlsE.* Recent studies have surprisingly shown that, with the exception of the RuvAB branch migrase, no other known recombination/repair proteins appear to play a role in the recombinational switching process. In the work presented here we show that G4 DNA can be formed by sequences within the B31 *vlsE* locus, prompting us to investigate the presence of potential G4-forming DNA throughout the *vls* locus of several Lyme spirochete strains and species. We found that runs of G, three nucleotides and longer occur at a very high density, with a greater than 100-fold strand-specific distribution in the *vls* locus of three *B. burgdorferi* strains as well as in *B. afzelii and B. garinii,* in spite of the bias for the use of A-T rich codons in *Borrelia* species. Our findings suggest the possibility that G4 DNA may be a mediator of recombinational switching at the *vlsE* locus in the Lyme spirochetes.

## Introduction

The host-pathogen interface represents a niche where accelerated genetic change is often required for pathogen survival and persistence. To avoid recognition and eventual destruction by an acquired immune response many bacterial, fungal and protozoal pathogens alter surface antigens through a process known as antigenic variation. This process, which imparts the ability to continually evade the host immune system, is often mediated through targeted gene rearrangements resulting in changes within the gene for an expressed surface antigen [1], [2], [3], [4]. In spite of the commonality of this pathogenic ruse, there has been limited progress in unraveling the mechanistic details of the recombination events underlying antigenic variation and how it is regulated in the almost three decades that it has been studied.

**Figure 1:**
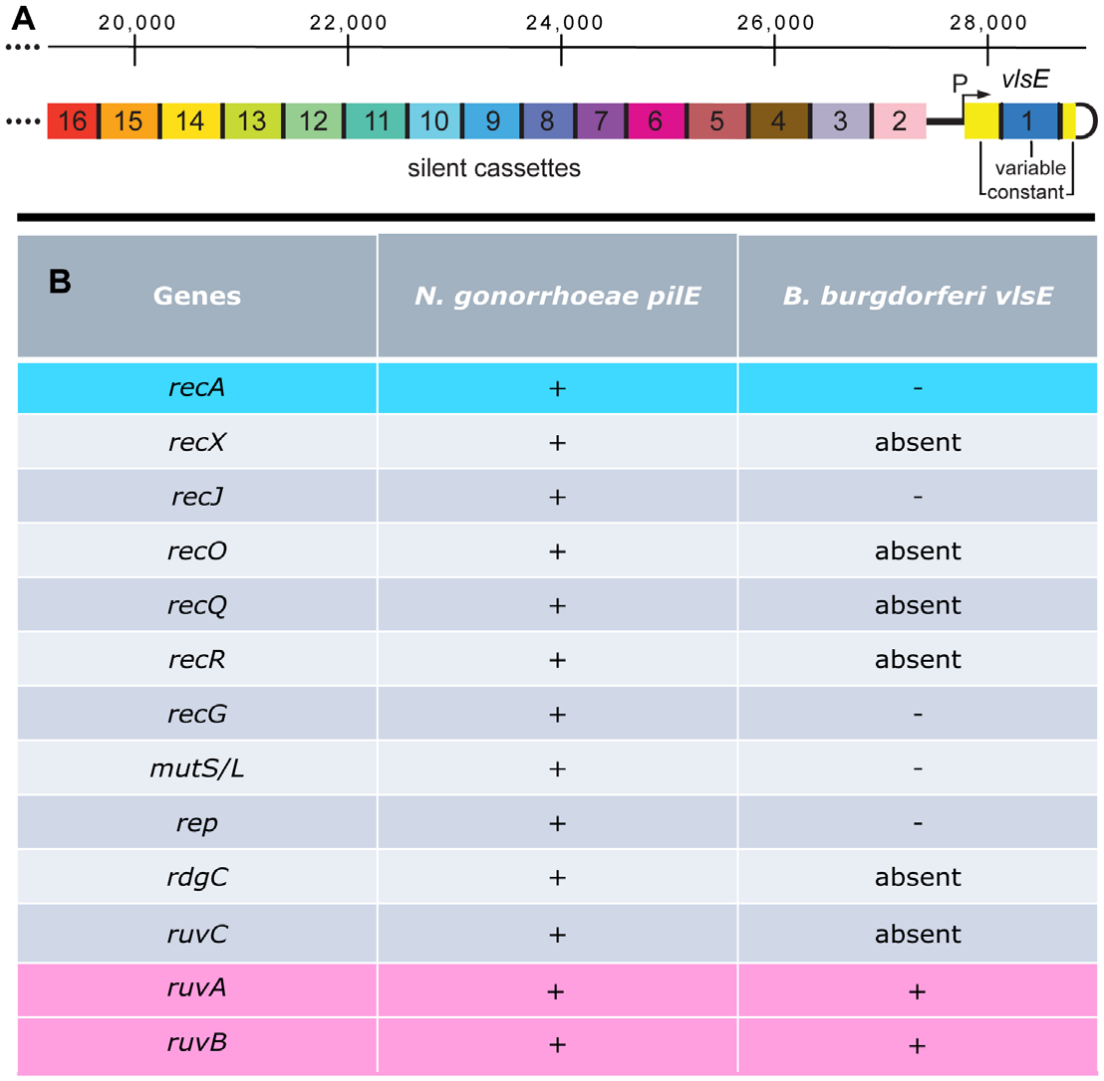
Schematic of antigenic variation in *B. burgdorferi*. **A)** Illustration showing the arrangement of the *vls* expression site (*vlsE*) and the contiguous array of 15 silent cassettes comprising the *vls* locus at the right end of the linear 28 kb plasmid, lp28-1 [8], [13]. *vlsE* is composed of a variable (blue) and two constant (yellow) regions separated by 17 bp direct repeats. The variable region is where DNA switching occurs. The 15 silent cassettes are displayed in different colors because they carry different variable regions. The silent cassettes are also separated by 17 bp direct repeats and are found in the opposite orientation of *vlsE.* Silent cassettes act as a source of DNA for nonreciprocal recombination events with the expression locus whereby segments within the *vlsE* variable region are replaced by sections of varied length from the silent cassettes. **B)** A comparison of the genes implicated in antigenic switching or its control at the *pilE* locus in *N. gonorrhoeae* [4], [15], [16], [17], [18], [19], [20], [21], [22], [23], [24], [25], [26], and the *vlsE* locus in *B. burgdorferi* [27], [28], [39]. A+sign denotes genes whose absence has a significant affect upon switching, while a – sign indicates genes that have no apparent involvement. The term “absent” indicates genes that are not present in the *B. burgdorferi* genome.

**Figure 2:**
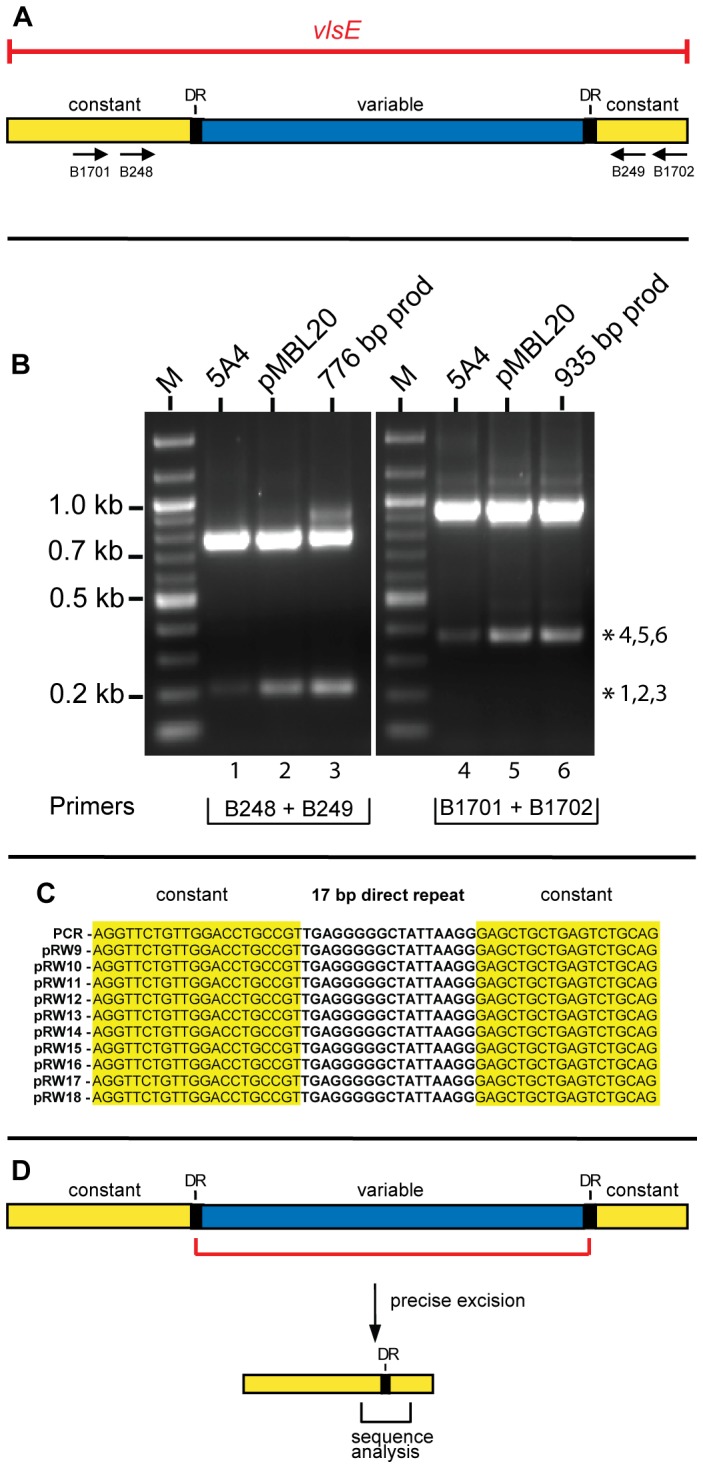
Precise excision of the *vlsE* variable region during PCR amplification. **A)** Schematic showing the variable, constant and 17 bp direct repeats of the *vlsE* gene. The location of PCR primers used for amplification are shown by arrows below the constant regions. **B)** An ethidium bromide-stained agarose gel showing amplification of a portion of *vlsE* with Phusion DNA polymerase using three different templates and two different primer sets. The templates used were *B. burgdorferi* B31 5A4 genomic DNA [29] pMBL20, a plasmid carrying the *vlsE* gene [51]; the 776 bp or 935 bp PCR products resulting from PCR amplification. The asterisks indicate smaller discrete bands observed in lanes 1–3 and 4–6. M denotes a 100 bp molecular weight ladder marker. PCR samples were run on a 1.2% agarose gel in 1X TAE buffer at 80 V for 1.2 hours. **C)** Characterization of precise deletions in *vlsE*. The first entry on the left side of the panel shows the sequence obtained from direct sequencing of the PCR product obtained with either B248 and B249 or B1701 and 1702. The remainder of the lineup shows sequence generated with 10 randomly selected *E. coli* transformants. The transformants were generated by cloning the 223 bp truncated PCR product generated *in vitro* with primers B248 and B249. The fragment was gel-excised and cloned into pJET1.2/blunt vector (Fermentas). The alignment shows that all the sequenced *vlsE* inserts had a precise excision of the 570 bp variable region. **D)** Schematic showing the precise excision of the *vlsE* variable region that occurred during PCR amplification.

Bacteria in the genus *Borrelia* cause Lyme borreliosis and relapsing fever (see [5]) and have efficient DNA rearrangement systems that promote antigenic variation, which results in persistent infection [6], [7]. **Figure** **1A** shows the antigenic variation system (the *vls* locus) of the Lyme spirochete *Borrelia burgdorferi* B31. The right end of the linear plasmid lp28-1 carries an expression site for VlsE, a surface lipoprotein. It also carries 15 silent cassettes that encode information corresponding to the *vlsE* variable region. The *vlsE* expression region is comprised of a central variable region that is flanked by constant regions (**Fig. 2A**). At the junction of the variable and constant regions are 17 bp direct repeat sequences [8]. These DR’s are also found between each of the silent cassettes, 2–16. DNA sequence is transferred unidirectionally from the silent cassettes to *vlsE* in a series of segmental gene conversions [8], [9], [10], [11], [12]. The resulting rearranged *vlsE* gene is a mosaic that can contain information from a number of the silent cassettes with a possibility to generate a myriad of VlsE variants (see [13]).

Recombinational switching at *vlsE* results in sequence variation of the VlsE protein that alters its antigenic properties and allows the spirochete to evade the antibody-mediated immune response. This recombinational switching only occurs during animal infection and does not occur in the tick gut or when the spirochetes are grown in culture [13], [14]. After inoculation of the mouse host, recombination events occur at a very high frequency, beginning as early as four days post-infection [10]. By 28 days post-infection none of the remaining spirochetes carry the *vlsE* sequence from the original infecting organisms [11].

Although switching at *vlsE* involves segmental gene conversion events, the molecular details of the recombination reaction remain uncharacterized. Surprisingly, of the 14 known recombination/repair genes (**Fig. 1B**) involved in the antigenic variation process in *Neisseria gonorrhoeae* [4], [15], [16], [17], [18], [19], [20], [21], [22], [23], [24], [25], [26], only the RuvAB branch migrase is required for switching at *vlsE* in *B. burgdorferi* [27], [28]. How then does *B. burgdorferi* promote this exotic recombination process in the apparent absence of the involvement of the usual cadre of recombination proteins?

In the work presented here we show that G4 DNA can be formed by DNA sequences within the B31 *vlsE* gene, alerting us to the possible presence of potential G4-forming DNA throughout the *vls* locus; G_3–5_ runs were found at a high density, with a strand–specific distribution, in spite of the bias for the use of A-T rich codons, in several Lyme *Borrelia* strains and species. The highly conserved profile of G-runs in the antigenic variation locus suggests a functional role, which we hypothesize may be involvement in the switching reaction through G4 DNA formation.

## Results

### Unusual Properties of the *vlsE* Locus of B31 during PCR Amplification

During routine PCR amplification of the *vlsE* region from *B.burgorferi* B31 5A4 [29] DNA using primers B248 and B249 (**Fig. 2A** **, Table S1**), we observed the appearance of a discrete ∼200 bp product along with the expected amplicon of 776 bp (**Fig. 2B**, lane 1). DNA sequencing revealed that the small fragment was a copy of the amplified region where a precise deletion of the *vlsE* variable region had occurred. Amplification using different templates or primers (**Fig. 2B**) or Taq polymerase instead of Phusion (data not shown) underscored the ubiquitous nature of the truncated PCR product. Cloning and sequencing of 10 randomly chosen *E.coli* transformants revealed an identical precise excision of 570 bp, which removed the entire *vlsE* variable region and one of the two 17 bp direct repeats (**Fig. 2C**). The truncated PCR fragment generated during PCR amplification corresponded to a precise deletion with a breakpoint within the two 17 bp direct repeats, as shown in **Fig. 2D**.

**Figure 3:**
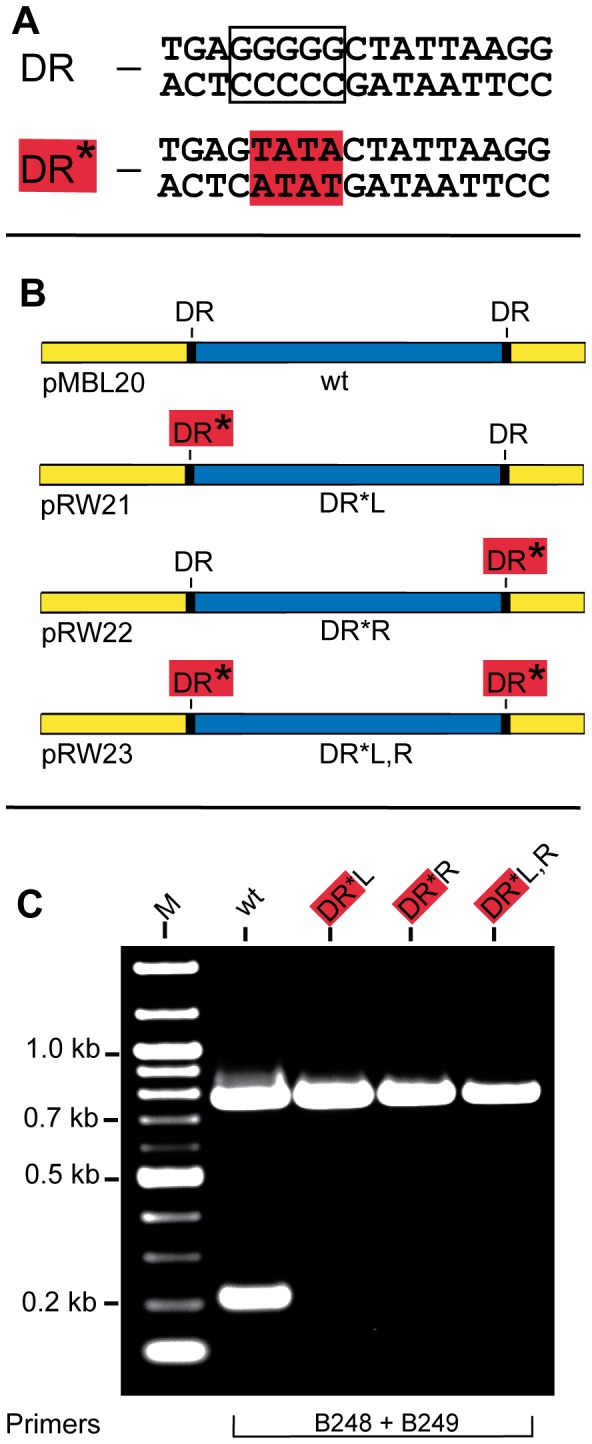
Affect of mutations in the 17 bp direct repeats on precise excision of the *vlsE* variable region. **A)** DNA sequences of the wild-type 17 bp direct repeat (DR) and a mutant 17 bp direct repeat (DR*) used in this study. Mutated bases are highlighted in red. **B)** Schematic showing the plasmid templates carrying wild-type DRs and a mutant DR at the left, right or both sides of the variable region. ***C*** **)** An ethidium bromide-stained agarose gel showing amplification of a portion of *vlsE* with Phusion DNA polymerase using the templates shown in Panel B with the indicated primers. Gel electrophoresis conditions were as noted in **Fig. 2**.

A closer look at the sequence of the17 bp DR’s showed a pentanucleotide G stretch (**Fig. 3A**). Such sequences are known to be involved in formation of G-quadruplex structures, also referred to as G4 DNA [30], [31], [32], [33], [34]. To directly probe the importance of the G run, site directed mutagenesis was used to generate constructs having a mutant direct repeat (DR*) at either the left, right or both sides (**Fig. 3B**) of the variable region by changing four nucleotides of the G stretch to TATA (**Fig. 3A**). The ability of the DR mutants to undergo precise excision was assayed by amplifying *vlsE* using primers B248 and B249. As expected, none of the DR mutants displayed precise excision of the *vlsE* variable region (**Fig. 3C**). The inability of the double mutant (DR*L,R) to restore precise excision indicates that the G stretch of the DR’s is important because the double mutant has two identical DR’s, but their sequence differs from the wild-type in the absence of the G run.

### Formation of Intermolecular G-quadruplex DNA by the DR’s of Strain B31

**Figure 4:**
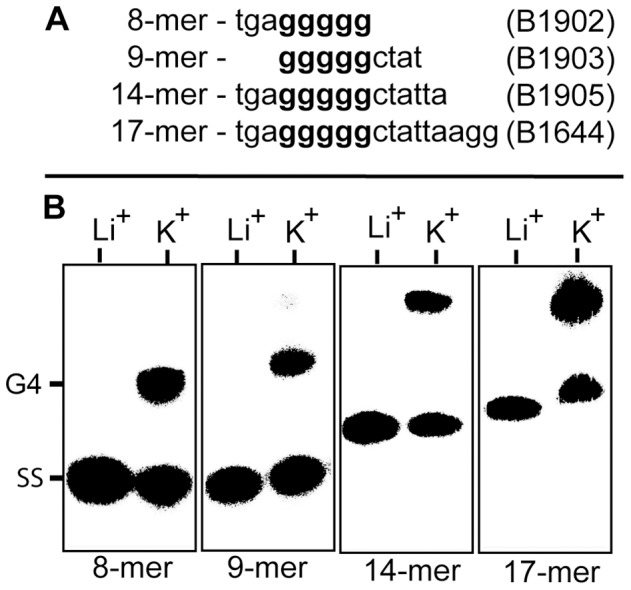
G-quadruplex (G4) formation by the *vlsE* 17 bp direct repeat. **A)** DNA sequences of the oligonucleotides used to assay for intermolecular G-quadruplex formation. The shortened oligos are all deleted forms of the full length 17-mer direct repeat. **B.** Electrophoretic mobility shift assay for G-quadruplex formation using 5′ ^32^P-labeled oligonucleotides from Panel A. The 5′ end-labeled oligos were annealed by heating at 95°C for 5 minutes followed by slow cooling for 15 hours to room temperature in presence of 200 mM LiCl or KCl. The products of the annealing reaction were run at 40 volts on a 10 cm, 20% native polyacrylamide gel in TBE buffer containing 25 mM K_2_B_4_O_7_ run at 4°C. Separated products were detected on a Perkin-Elmer Cyclone phosphorimager. SS represents single stranded oligonucleotide and G4 indicates the position of intermolecular G-quadruplex DNA.

**Figure 5:**
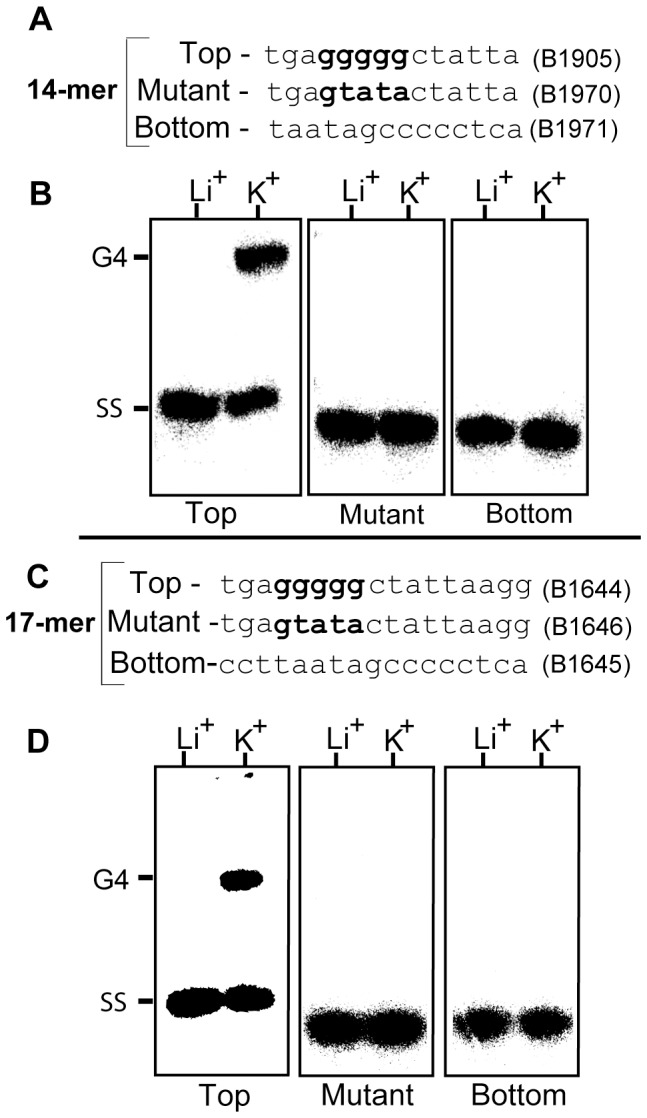
The affect of DNA strand and mutations in the G–motif on quadruplex formation. **A,C)** Oligonucleotides corresponding to the wild-type 14-mer and 17-mer top (see **Fig. 4**) and bottom strands, and mutated top strands in which four successive G’s were substituted with TATA. **B,D)** Autoradiogram of a 20% native gel run to assess G4 formation, as described in **Fig. 4**.

To directly probe the ability of the 17 bp DR’s to participate in G-quadruplex formation we synthesized the top (G-rich) strand of the 17 bp DR as well as several deleted forms of the oligo (**Fig. 4A**). The oligos were labeled at their 5′ ends and used in gel shift assays to monitor their ability to form G4 DNA (**Fig. 4B**). The oligonucleotides were self annealed either in 200 mM KCl to promote G4 formation or in 200 mM LiCl to inhibit it. All four oligos formed about 50% G4 DNA based upon reduced gel mobility in the presence of K^+^ but not Li^+.^ The pentanucleotide G stretch appeared to be the only region required for G4 formation. To further probe the role of the G run in quadruplex formation, we incorporated the previously used DR* mutations (see **Fig. 3A**) in the G-motif of the top strands of the 14-mer and the 17-mer by substituting four successive G’s with TATA (**Fig. 5A, C**). We also synthesized the bottom strands of the 14-mer and 17-mer. Neither the mutant top strands nor the C-rich bottom strands formed G4 DNA (**Fig. 5B, D**).

**Figure 6:**
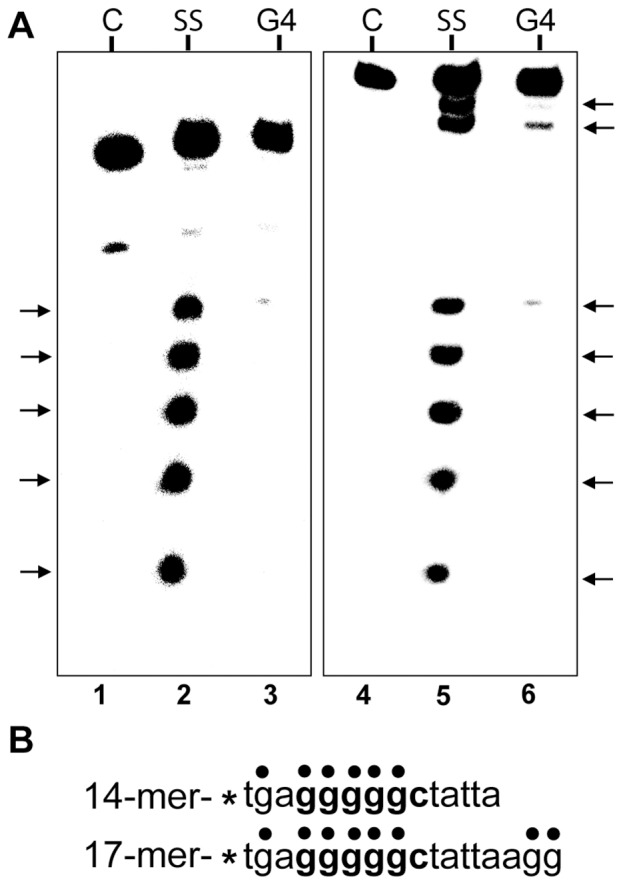
Methylation protection of the *vlsE* G4-forming sequence. **A)** Autoradiogram of a 25% denaturing polyacrylamide gel of dimethylsulfate treated 14-mer and 17-mer oligonucleotides. ^32^P-labeled oligonucleotides were annealed in 200 mM KCl and treated with 0.5% dimethylsulfate. The methylated oligos were then subjected to electrophoresis in a 20% native polyacrylamide gel to separate the single stranded (SS) oligos from the G4-DNA. The free and the G4 bands were treated with 1 M piperidine to induce strand breaks at the methylated guanine residues and the cleavage products were resolved on a 25% denaturing polyacrylamide gel. C represents the control oligo which was not treated with DMS. The arrows correspond to the DMS-protected guanine residues. ***B)*** Sequence of the oligonucleotides used in this experiment. The dots show the position of G residues. The smallest cleavage fragment was run off the bottom of the gel.

To confirm that the slower migrating gel bands were indeed G4 DNA, dimethyl sulfate (DMS) protection assays [35] were performed with the 14-mer and 17-mer DR. In a G-quadruplex the N7 position of the guanines are involved in Hoogsteen bonding and are inaccessible to methylation by DMS. The oligos were annealed in 200 mM KCl and methylated using 0.5% DMS. The methylated oligos were run on a 20% native polyacrylamide gel (as shown in **Fig. 6**) and the upper (G4) and lower (single stranded or SS) bands were excised and treated with 1 M piperidine to induce strand breaks at the methylated guanine residues. End-labeled cleavage fragments were resolved on a 25% denaturing polyacrylamide gel (**Fig. 6A**). Lanes 1 and 4 show the untreated 14-mer and 17-mer oligonuceotides, respectively. Lanes 2 and 5 show the DMS cleavage pattern of the faster migrating (lower gel band) single stranded oligo monomers while lanes 3 and 6 show the footprint of the slower moving G4 bands. In lanes 3 and 6 the N7 positions within the pentanucleotide G runs were protected from DMS modification, as expected for G-quadruplex. Interestingly, the two G residues at the 3′ end of the 17 bp DR were also protected, suggesting that they were also involved in the formation of G-tetrads (**Fig. 6A, B**). A G4 synapse of 17 bp DR’s could promote precise deletion *in vitro*, as shown in **Fig. 2** and **3**, by promoting premature termination of DNA synthesis followed by use of the incomplete product to prime DNA synthesis in a subsequent cycle.

### An Unprecedented Distribution of G-runs on the Coding Strand of *vls* DNA

**Figure 7:**
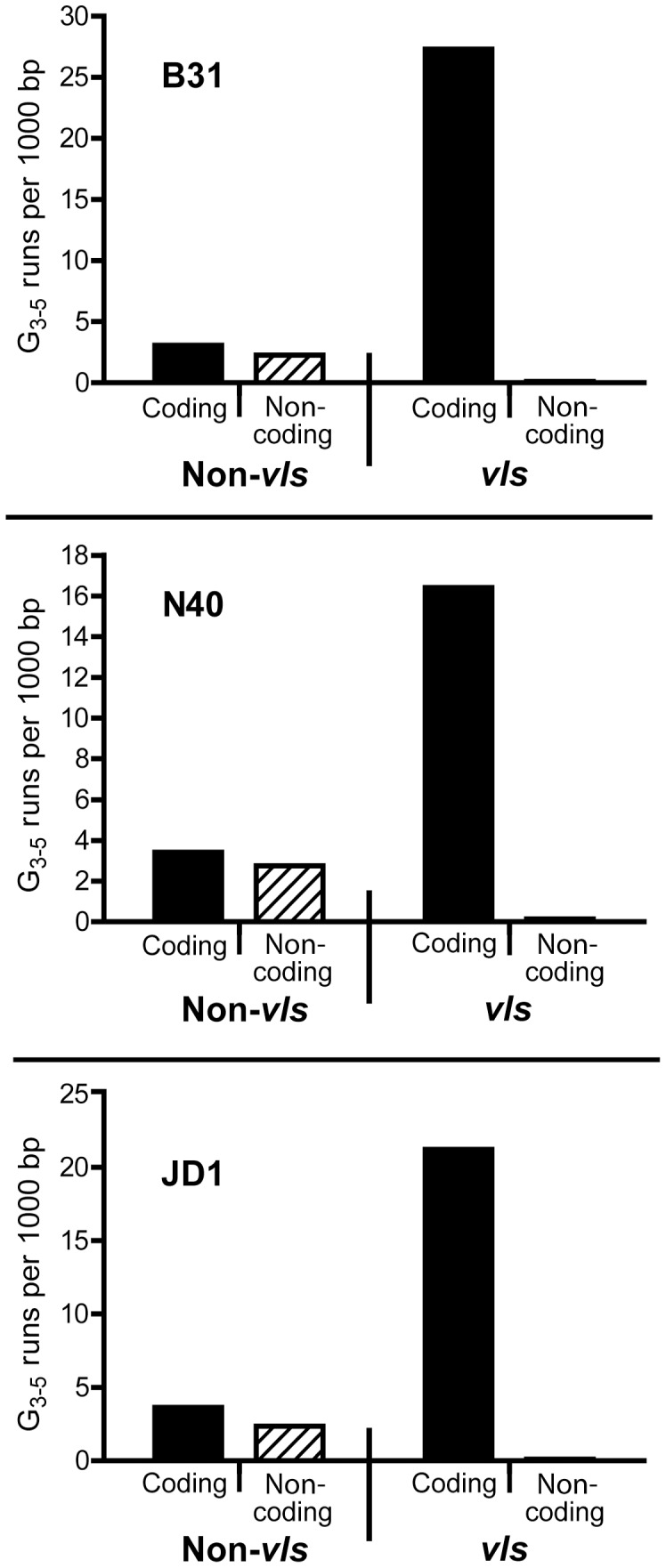
Distribution of G_3–5_ runs on the linear plasmids carrying the *vls* loci in *B. burgdorferi* strains B31, N40 and JD1. G runs of 3–5 nucleotides in length were counted in the sequence of lp28-1 (Accession NC_001851 and FJ472338) from B31 [8], [52], [53], lp36 (Accession CP002230) from N40 and lp28-1 (Accession NC_017404) of JD1 [36]. The distribution of G_3–5_ runs on both strands of *vls* and non-*vls* DNA was plotted. The coding strand is defined as the strand in which the silent cassettes code for the VlsE protein. The *vls* DNA includes only the silent cassettes, as the *vlsE* sequence is only known for B31.

G-runs are not present in the DR’s of other Lyme spirochetes and the DR’s are not believed to be involved in antigenic switching at *vlsE* (see Discussion). However, their ability to confer unusual properties upon the B31 *vlsE* locus *in vitro*, presumably through the formation of G4 DNA, prompted us to investigate the potential for formation of G4 DNA throughout the *vls* locus in several Lyme spirochetes. We analyzed the sequence of the linear plasmids carrying the *vls* locus from *B. burgdorferi* B31, N40 and JD1 [8], [36] for runs of G that were three or longer. These three strains show similar levels of divergence from one another with about 65% identity between *vls* cassettes [37] and contain clearly different DR’s within the silent cassettes [38]. Analysis of the B31 plasmid lp28-1, which carries the *vls* locus (**Fig. 7**), showed the presence of 2–3 G-runs per 1000 bp on either strand in the first 19,000 bp at the left end of the plasmid. In dramatic contrast, the *vls* region itself (8,239 bp) contained a total of 225 G-runs on the coding strand (27 runs per kb) and only one on the non-coding strand. This grossly unequal weighting of G-runs on the *vls* coding strand was also apparent on lp36 in strain N40 and on lp28-1 in strain JD1 (**Fig. 7**). Moreover, the somewhat more divergent *vls* regions from *B. garinii* Ip90 and *B. afzelii* ACA1 [38] also displayed 26.6 and 21.1 G-runs per thousand bp of *vls* coding strand, respectively, with no G-runs present on the non-coding strand. The conservation of this unprecedented distribution of G-runs on the *vls* coding strand between strains and between Lyme borriliae strongly argues for a functional role – especially when considering that G-rich codons are infrequently used in the A-T rich *Borrelia* genomes. The GGG glycine codon so prevalent within the G-runs is the least commonly used of the four (GGX) glycine codons in *B. burgdorferi.*

## Discussion

Antigenic variation through DNA rearrangements provides a powerful diversity generating mechanism for pathogens to evade the host acquired immune response. In spite of the commonality of this pathogenic tactic, the molecular details underlying the DNA shuffling process remain largely uncharacterized. The most well studied system is the bacterial pathogen *N. gonorrhoeae* (see [4], [15]). This organism uses a large collection of recombination/repair proteins, including RecA, to promote the necessary gene conversion events. In contrast, *B. burgdorferi* does not require RecA for switching at *vlsE* [27], [39] and the only required factor identified thus far is the RuvAB branch migrase [27], [28]. It is an enigma as to how such a complex DNA rearrangement system can be mediated so efficiently by *B. burgdorferi* with such a paucity of protein factors. One possible explanation is that as yet identified specialized proteins(s) are the predominant player(s) in promoting the reaction. The widely varying positions of the observed *vlsE* breakpoints and the highly variable sizes of the exchanged DNA segments [11] are clearly not consistent with the activities of any known types of site-specific recombinases (eg serine and tyrosine recombinases). However, specialized proteins with alternative recombinational activities remain a distinct possibility. Yet another prospect is that recombinational switching at *vlsE* occurs through a simplified pathway requiring fewer protein factors but employing unusual DNA structures.

The 17 bp DR’s were originally suggested as likely DNA sites involved in recombinational switching at *vlsE* in *B. burgdorferi* B31 [8]. However, subsequent sequence analysis of *vls* regions from other Lyme *Borrelia* species and strains [38] revealed that the 17 bp DR’s, although well conserved within the B31 *vls* locus, are not well conserved across species or even within *B. burgdorferi* strains. Moreover, the *B. burgdorferi* strain N40 has two different DR’s of 15 and 18 bp and has a total of only 9 DR’s in 20 *vls* cassettes [36]. This lack of conservation in DR sequence and arrangement argues against a general role for the DR’s in recombinational switching; we do not believe that the *in vitro* precise deletion reaction involving the DR’s that we report here has any direct relevance to the mechanism of switching at *vlsE*. However, the ability of the B31 DR’s to form G4 DNA prompted us to look for potential G4-forming DNA throughout the *vls* locus. Our analysis of DNA sequence from three *B. burgdorferi* strains (B31, N40 and JD1) as well as two other Lyme *Borrelia* species (*B. afzelii* and *B. garinii*) revealed a striking number and distribution of G-runs on the *vls* coding strand in all cases (**Fig. 7**), suggesting the possibility that the formation of G4 DNA might in fact be an important feature involved in recombinational switching and antigenic variation in Lyme borriliae.

While the literature regarding the formation and characterization of G-quadruplexes *in vitro* is extensive (see [31], [32], [33]), information on the *in vivo* relevance of such structures has lagged behind. However, evidence is accumulating for the *in vivo* existence of G-quadruplex DNA [40], [41], [42] and for diverse biological roles for G4 structures in the regulation of telomere maintenance, replication, transcription and translation (see [31], [32], [33], [34]). Unusual DNA structures such as G4 DNA may have a profound effect upon genome stability [43] and potential G4 forming DNA sequences have been shown to be enriched at recombinational hotspots [43], [44], [45]. Of particular relevance, recent work on pilin antigenic variation in *N. gonorrhoeae* has revealed a role for G-quadruplex in initiating the recombination reaction [44], [45]. In this system a 16 bp G-rich sequence forms an intramolecular G4 structure near the pilin locus. This G-quadruplex is required as a signal for nicking. The nicks are subsequently processed by the RecJ exonuclease, the RecQ and Rep helicases and the RecA protein. While this mechanism is clearly quite different (based upon required protein factors) than the mechanism used by *B. burgdorferi,* it nonetheless indicates a clear *in vivo* role for G4 DNA in modulating a bacterial recombination reaction.

Before ensuing with further discussion we need to note that G4 DNA normally occurs within a short stretch of DNA and that the G-runs in the *vls* locus are not closely juxtaposed. Nonetheless, G4 DNA can be assembled by inter-molecular association *in vitro*, as shown in **Fig. 4** **,** **5** **,** **6**, and is believed to form *in vitro* between plasmid-borne DR’s flanking the *vlsE* variable region resulting in the precise deletion observed in **Fig. 2** and **3**. *In vivo,* formation of G4 DNA would require G-runs in single stranded DNA and single stranded regions are known to be generated during processes such as transcription and replication, which involve DNA unwinding.

In view of the growing importance of G4 DNA in biological functions and the very biased distribution of G-runs on the *vls* coding strand, how might G-quadruplex DNA function in recombinational switching at *vlsE*? There are three important properties that G4-forming sequences might contribute: The first is that a large number of G-runs in a piece of DNA could provide a type of “molecular velcro” for bringing together the *vlsE* gene and the silent cassettes to promote DNA exchange by an as yet characterized molecular mechanism. With 17 G-runs in the variable region of *vlsE* in B31 and 225 in the silent cassettes, one can imagine a large network of potential associations to form G-quadruplex. The second is that DNA replication is potently inhibited by G4 DNA, especially in the leading strand [41]. Interestingly, in *vlsE* in B31, N40 and JD1, the G-runs are synthesized on the leading strand while in the silent cassettes they are generated on the lagging strand. It is tempting to speculate that this arrangement may influence the overall directionality of the gene conversion reaction. The third is that the G4 structure (or stalled replication fork) may provide a specific target for subsequent strand exchange by acting as a site for DNA cleavage [44], [46], binding of specialized proteins [47] or polymerase template strand switching [48].

Our current inability to genetically manipulate the *vls* locus in *B. burgdorferi,* coupled with the requirement of mouse infections to study switching at *vlsE* precludes the direct testing of a role for G4 DNA in this process at present. Nonetheless, our studies establish the ability of the G-runs in the B31 bp *vlsE* gene of *B. burgdorferi* B31 to self-synapse *in vitro.* The dramatic, strand-specific preponderance of high numbers of G-runs within the *vls* locus of several strains and species supports a model where G-quadruplex DNA may play an important role in the recombinational switching process. Further studies will be required to test this hypothesis.

## Materials and Methods

### Bacterial Strains and Culture Conditions

*E. coli* strains used in this study (**Table S1**) were DH5α and SURE 2 (Stratagene). All *E. coli* strains were grown in Luria Broth. DH5α clones were grown at 37°C, while SURE 2 clones were incubated at 30°C. Infectious *Borrelia burgdorferi* 5A4, derived from the type strain B31 [29] was used as the parental strain to generate wt *vlsE* DNA and was cultivated at 35°C (with a 1.5% CO_2_ environment) in BSK-II medium supplemented with 6% rabbit serum (Cedarlane Laboratories, Burlington, ON, CA) and prepared in-house [49]. Bacterial density was determined using a Petroff- Hausser Chamber (Hausser Scientific Partnership) under dark-field.

### PCR Screening Assays and Cloning of PCR Products

The presence or absence of the *vlsE* variable region was initially screened with the PCR primers, B248 and B249 or B1701 and B1702 (**Table S2**) using Phusion DNA polymerase (Finnzymes) and Taq DNA polymerase (New England Biolabs). Reaction conditions were as follows: 94°C for 2 minutes, 25 cycles of 94°C for 30 seconds, 50°C for 30 seconds and 68°C for 1 minute, followed by a final extension of 68°C for 7 minutes. PCR reactions were analyzed on 1.2% agarose gels in 1X TAE buffer at 80 V for 1.5 hours. The gels were stained with ethidium bromide to allow visualization. For cloning, the PCR products were excised and gel purified using the Qiagen Gel Extraction Kit. Purified fragments were cloned into the pJET1.2/blunt vector (CloneJet, Fermentas) and used to transform *E.coli* DH5α or SURE 2 cells when the large inverted repeat in the *vlsE* promoter region was present. The transformations were plated on LB agar plates containing 100 µg/ml carbenicillin at 37°C. For DNA sequencing, 10 transformants were picked and grown overnight in five ml LB supplemented with 100 µg/ml carbenicillin. Plasmid DNA was isolated using the Qiagen plasmid miniprep kit and sequenced by the University of Calgary Core DNA Services using the pJET1.2 forward/Custom sequencing primer (CloneJet, Fermentas).

### Site-directed Mutagenesis

Site-directed mutagenesis was performed as previously reported [50]. Generation of mutations in the right-end 17 bp direct repeat was carried out using pMBL20 [51] as a template and the primers B1195 and B1196 (**Table S2**). The left-end and double DR mutants were generated by using pMBL20 and pRW22, respectively as templates for site-directed mutagenesis using the PCR primers, B1241 and B1242. Mutations were confirmed through DNA sequencing. PCR conditions for the 50 µl reactions were as follows: 1X Phusion High Fidelity (GC) buffer (New England Biolabs), 3% DMSO, 0.1 mM dNTPs, 0.02 units/µl Phusion DNA polymerase (New England Biolabs), 0.4 pmol/µl of each and 1.5 ng/µl plasmid DNA template. The first three PCR cycles were performed separately for each primer (e.g. one tube for F primer, one tube for R primer). After the third cycle the contents of the different primer tubes were mixed, and PCR cycling was continued to completion. PCR cycling conditions were as follows: 98°C 45 seconds followed by 25 cycles of 98°C, 20 seconds; 55°C, 30 seconds; 72°C, 3.5 minutes and then 72°C for 7 minutes. PCR products were purified using the QIAQuick PCR purification kit, according to the manufacturer’s instructions (Qiagen), and then template DNA was digested with *DpnI* (New England Biolabs). Purified, digested DNA was used directly to transform chemically competent DH5α.

### End-labeling of Oligonucleotides

The 5′-end-labeled single-strand oligonucleotides were obtained by incubating the oligonucleotides with T4 polynucleotide kinase (New England BioLabs) and [γ-^32^P]ATP for 30 minutes at 37°C using manufacturer specified conditions. Labeled oligonucleotides were purified with a Microspin G-25 Sephadex column (GE Healthcare) after inactivation of the kinase by heating at 65°C for 20 minutes.

### G4 DNA Electrophoretic Mobility Shift Assay

The 5′ ^32^P-labeled DNA oligos (24 pmoles) were self annealed in 200 mM KCl or LiCl by heating at 95°C for 5 minutes in a heating block and then cooled slowly to room temperature at a rate of 0.025°C per second and further incubated at room temperature for 15 hours. The annealed products were run on a 10 cm −20% native polyacrylamide gel prepared in TBE buffer containing 25 mM K_2_B_4_O_7._ The gel was run at 40 volts for 8 hours at 4°C. The gel was transferred onto 3 MM Whatman paper and covered with saran wrap. The gel was dried in a dryer at 65°C for 1 hour. An autoradiogram was obtained by exposing the gel to phosphorimager screen overnight. Separated products were detected on a Perkin-Elmer Cyclone phosphorimager.

### Dimethyl Sulfate Protection

Gel-purified oligos were end labeled with [γ-^32^P] ATP and annealed in the presence of 200 mM KCl as described earlier. Each annealed DNA was treated with 0.5% dimethyl sulfate (DMS) for 30 minutes at room temperature to methylate the DNA. DMS modification reactions were stopped by adding one-tenth volume of gel loading buffer containing 1 µg of lambda DNA, and the modification products were immediately loaded onto a 20% native polyacryamide gel to separate free single stranded oligo from intermolecular quadruplexes. The gels were run in 1XTBE buffer with 25 mM K_2_B_4_O_7_ at 40 volts for 8 hours at 4°C_._ Single stranded and G4 DNA was eluted from the gel, soaked overnight in TE buffer at 37°C and passed through a G10 Sephadex syringe column. The samples were dried in a speed vac and the pellet was resuspended in 100 µl of 1 M piperidine and incubated at 95°C for 30 minutes. The samples were dried in a speed vac followed by washing of the pellets with 100 µl water. The samples were dried again in a speed vac and the DNA pellets were resuspended in 20 µl of alkaline sequence loading dye. The cleaved products were resolved on a 25% denaturing polyacrylamide gel. The gel was allowed to polymerize overnight and was pre-run at 600 volts for 2 hours. The samples (10 µl) were heated at 95°C for 3 minutes and run at 2000 volts for 3.5 hours on a 40 cm sequencing gel. The gel was transferred onto 3 MM Whatman paper and covered with saran wrap. An autoradiogram was obtained by exposing the gel to the phosphorimager screen overnight.

## Supporting Information

Table S1 Primers and oligonucleotides used in this study.
(DOC)

Table S2 DNA plasmids and *E. coli* strains used in this study.
(DOCX)
