# Supplementary material for: Suggested Role for G4 DNA in Recombinational Switching at the Antigenic Variation Locus of the Lyme Disease Spirochete
Source: PLoS One. 2013 Feb 28;8(2):e57792. doi: 10.1371/journal.pone.0057792 (PMC3585125; doi:10.1371/journal.pone.0057792)
Supplement: Table S1 — Primers and oligonucleotides used in this study. (DOC) [file pone.0057792.s001.doc]

**Table S1. Primers and oligonucleotides** used in this study.

| Primer | Sequence (5’ – 3’) | Use |
| --- | --- | --- |
| B248 | GCGATATAAGTAGTACGACGGGGAAACCAG | PCR screen for *vlsE* variable region and DNA sequencing |
| B249 | CAAGGCAGGAGGTGTTTCTTTACTAGCAGC |
| B1701 | TGGGTTAGTAGCAGAGGCTTTTGG |
| B1702 | GTCCATACACTTAATTAAATCACTTATT |
| B1195 | GAAAGAGAAGGCTGAGTATACTATTAAGGGAGCTGCTGAG | Site-directed mutagenesis of right 17 bp direct repeat |
| B1196 | CTCAGCAGCTCCCTTAATAGTATACTCAGCCTTCTCTTTC |
| B1241 | GTTGGAACTGCCGTTGAGTATACTATTAAGGAAGTTAGCGAGTTG | Site-directed mutagenesis of left 17 bp direct repeat |
| B1242 | CAACTCGCTAACTTCCTTAATAGTATACTCAACGGCAGTTCCAAC |
| B1644 | TGAGGGGGCTATTAAGG | 17-mer DR top strand  17-mer bottom strand |
| B1645 | CCTTAATAGCCCCCTCA |
| B1646 | TGAGTATACTATTAAGG | 17-mer mutant top strand |
| B1902 | TGAGGGGG | 8-mer top strand |
| B1903 | GGGGGCTAT | 9-mer top strand |
| B1905 | TGAGGGGGCTATTA | 14-mer top strand |
| B1970 | TGAGTATACTATTA | 14-mer mutant top strand |
| B1971 | TAATAGCCCCCTCA | 14-mer bottom strand |
|  |  |  |
|  |  |  |
