# Supplementary material for: Suggested Role for G4 DNA in Recombinational Switching at the Antigenic Variation Locus of the Lyme Disease Spirochete
Source: PLoS One. 2013 Feb 28;8(2):e57792. doi: 10.1371/journal.pone.0057792 (PMC3585125; doi:10.1371/journal.pone.0057792)
Supplement: Table S2 — DNA plasmids and E. coli strains used in this study. (DOCX) [file pone.0057792.s002.docx]

**Table S2. DNA plasmids and *E. coli* strains used in this study.**

| **Plasmid** | **Strain / source** | **Description/Purpose** |
| --- | --- | --- |
| pJET1.2/blunt | Fermentas | vector |
| pMBL20 | GCE2035 | Fig. 2 Plasmid template |
| pRW9 | GCE2017 | Fig. 2 pJET clone, deletion generated *in vitro* |
| pRW10 | GCE2018 | Fig. 2 pJET clone, deletion generated *in vitro* |
| pRW11 | GCE2019 | Fig. 2 pJET clone, deletion generated *in vitro* |
| pRW12 | GCE2020 | Fig. 2 pJET clone, deletion generated *in vitro* |
| pRW13 | GCE2021 | Fig. 2 pJET clone, deletion generated *in vitro* |
| pRW14 | GCE2022 | Fig. 2 pJET clone, deletion generated *in vitro* |
| pRW15 | GCE2023 | Fig. 2 pJET clone, deletion generated *in vitro* |
| pRW16 | GCE2024 | Fig. 2 pJET clone, deletion generated *in vitro* |
| pRW17 | GCE2025 | Fig. 2 pJET clone, deletion generated *in vitro* |
| pRW18 | GCE2026 | Fig. 2 pJET clone, deletion generated *in vitro* |
| pRW21 | GCE2029 | Fig.3 Left 17bp DR mutant |
| pRW22 | GCE2031 | Fig.3 Right 17bp DR mutant |
| pRW23 | GCE2033 | Fig.3 double 17bp DR mutant |
